# Cardioprotective Properties of Omentin-1 in Type 2 Diabetes: Evidence from Clinical and *In Vitro* Studies

**DOI:** 10.1371/journal.pone.0059697

**Published:** 2013-03-29

**Authors:** Sabrina Greulich, Weena J. Y. Chen, Bujar Maxhera, Luuk J. Rijzewijk, Rutger W. van der Meer, Jacqueline T. Jonker, Heidi Mueller, Daniella Herzfeld de Wiza, Ralf-Ruediger Floerke, Konstantinos Smiris, Hildo J. Lamb, Albert de Roos, Jeroen J. Bax, Johannes A. Romijn, Jan W. A. Smit, Payam Akhyari, Artur Lichtenberg, Juergen Eckel, Michaela Diamant, D. Margriet Ouwens

**Affiliations:** 1 Institute of Clinical Biochemistry and Pathobiochemistry, German Diabetes Center, Duesseldorf, Germany; 2 Diabetes Center, Department of Internal Medicine, VU University Medical Center, Amsterdam, The Netherlands; 3 Department of Cardiovascular Surgery, Medical Faculty, Heinrich-Heine-University, Duesseldorf, Germany; 4 Department of Radiology, Leiden University Medical Center, Leiden, The Netherlands; 5 Department of Endocrinology, Leiden University Medical Center, Leiden, The Netherlands; 6 Department of Cardiology, Leiden University Medical Center, Leiden, The Netherlands; 7 Department of Medicine, Academic Medical Center, University of Amsterdam, The Netherlands; 8 Paul Langerhans Group, German Diabetes Center, Duesseldorf, Germany; 9 Department of Endocrinology, Ghent University Hospital, Ghent, Belgium; University of Padova, Medical School, Italy

## Abstract

**Context:**

Adipokines are linked to the development of cardiovascular dysfunction in type 2 diabetes (DM2). In DM2-patients, circulating levels of omentin-1, an adipokine preferentially expressed in epicardial adipose tissue, are decreased. This study investigated whether omentin-1 has a cardioprotective function.

**Methods:**

Omentin-1 levels in plasma and cardiac fat depots were determined in DM2-patients versus controls. Moreover, the relation between omentin-1 levels and cardiac function was examined in men with uncomplicated DM2. Finally, we determined whether omentin-1 could reverse the induction of cardiomyocyte dysfunction by conditioned media derived from epicardial adipose tissue from patients with DM2.

**Results:**

Omentin-1 was highly expressed and secreted by epicardial adipose tissue, and reduced in DM2. Circulating omentin-1 levels were lower in DM2 versus controls, and positively correlated with the diastolic parameters early peak filling rate, early deceleration peak and early deceleration mean (all *P*<0.05). The improved diastolic function following pioglitazone treatment associated with increases in omentin-1 levels (*P*<0.05). *In vitro*, exposure of cardiomyocytes to conditioned media derived from epicardial adipose tissue from patients with DM2 induced contractile dysfunction and insulin resistance, which was prevented by the addition of recombinant omentin.

**Conclusion:**

These data identify omentin-1 as a cardioprotective adipokine, and indicate that decreases in omentin-1 levels could contribute to the induction of cardiovascular dysfunction in DM2.

## Introduction

Cardiac dysfunction and myocardial insulin resistance are common in patients with type 2 diabetes (DM2) [1], [2]. Recent studies have implicated adipokines in the pathogenesis of these cardiac alterations [3]–[6]. Omentin-1 is an adipokine with decreased circulating levels in conditions associated with insulin resistance [7]–[12]. Plasma omentin-1 levels inversely correlate with body mass index (BMI), fat mass, and fasting plasma insulin, and positively with insulin sensitivity, adiponectin, high density lipoprotein cholesterol, and endothelial function [8], [10], [11], [13], [14]. *In vitro*, omentin-1 enhances insulin-mediated stimulation of Akt-phosphorylation, glucose uptake and inhibits tumor necrosis factor α-induced inflammation [15], [16]. This indicates that omentin-1, like adiponectin, may act as a protective adipokine.

Omentin-1 is abundantly expressed in epicardial adipose tissue (EAT), a visceral fat depot located around the heart and coronary arteries [5], [17]. Since EAT is not separated by a fascia from the myocardium, factors secreted from EAT may directly affect cardiac function [3], [5], [18], [19]. We examined whether omentin-1 expression and secretion in various adipose tissue depots, including EAT, pericardial, and subcutaneous adipose tissue, is altered in patients with DM2. Furthermore, we evaluated whether omentin-1 levels were associated with metabolic and cardiac parameters in men with uncomplicated DM2 before and after intervention with the insulin-sensitizing drug pioglitazone, which is known to act predominantly on adipose tissue and was shown to improve cardiac diastolic function in this cohort [20], [21]. Finally, we examined whether recombinant omentin-1 prevents the abrogation of insulin action and induction of contractile dysfunction in primary rat cardiomyocytes by conditioned media derived from EAT from patients with DM2.

## Materials and Methods

### Patient Characteristics

The patient samples used in this study were collected from two distinct cohorts.

At the University Hospital in Duesseldorf (Germany), Caucasian men undergoing coronary artery bypass grafting with or without additional valve replacement surgery consented to the collection of biopsies from EAT, pericardial, and intrathoracic subcutaneous adipose tissue after written informed consent. The procedure to obtain adipose tissue samples was approved by the medical ethical committee of the Heinrich-Heine-University (Duesseldorf, Germany), and the study was conducted in accordance with the principles of the Declaration of Helsinki. Patients of other ethnic origins, diagnosed as having human immunodeficiency virus infection, lipodystrophy or chronic coexistent inflammatory disease were excluded from participation. Participants were distributed into two groups, i.e. those with and those without DM2, on the basis of the diagnosis ‘DM2’ in the medical record of the patient. Adipose tissue biopsies were either used to generate conditioned media (CM), or snap-frozen in liquid nitrogen and stored at −80°C for protein extraction.

Circulating omentin-1 levels were determined in 78 men with uncomplicated DM2 from the previously described PIRAMID (Pioglitazone Influence on tRiglyceride Accumulation in the Myocardium in Diabetes) study [22] and 14 healthy men, aged 45–65 years, with normal glucose metabolism as determined by a 75-g oral glucose tolerance test, which served as control subjects [23] (Clinical trial registration number (unique identifier): ISRCTN53177482; URL: http://www.controlled-trials.com/ISRCTN53177482). Participating healthy controls and men with uncomplicated DM2 had a body mass index (BMI) between 25–32 kg/m^2^, and a blood pressure lower than 150/85 mm Hg (with or without the use of antihypertensive drugs). Furthermore, only DM2 patients with glycosylated hemoglobin (HbA1c) between 6.5–8.5% were eligible for inclusion. Patients with DM2 diagnosed as having any symptoms or history of diabetes-related complications, or cardiovascular or liver disease, as well as prior use of thiazolidinediones or insulin, were excluded from participation. Exclusion criteria for healthy controls were a history or current cardiovascular disease, dyslipidemia, and the use of any prescribed medication. The clinical studies were conducted at two university medical hospitals in the Netherlands (Leiden University Medical Center, Leiden, and VU University Medical Center, Amsterdam), were approved by the medical ethics committee of both centers, and performed in full compliance with the Declaration of Helsinki. Eligible participants with DM2 ceased their regular blood glucose lowering agents before entering a 10-week run-in period in which they were transferred to glimepiride monotherapy and titrated until a stable dose was reached 2 weeks before randomization. Then, participants with DM2 were randomized to pioglitazone (15 mg once daily, titrated to 30 mg once daily after 2 weeks) or metformin (500 mg twice daily, titrated to 1000 mg twice daily) in addition to glimepiride throughout the study. Details on assessment of cardiac function, aortic pulse wave velocity and distensibility, and volume of pericardial and visceral abdominal adipose tissue in the study groups have been described elsewhere [21]–[24]. Insulin sensitivity was assessed by a euglycemic hyperinsulinemic clamp using an insulin infusion rate of 40 mU.m^−2^.min^−1^ as previously described [25], [26]. Fasting plasma samples for determination of omentin-1 levels were collected before randomization and in the case of participants with DM2 after 24 weeks of pioglitazone or metformin therapy.

### Generation of Conditioned Media

CM was generated as described previously [3], [27], [28]. Briefly, freshly collected adipose tissue biopsies were washed 3 times with phosphate-buffered saline (PBS), supplemented with antibiotic-antimycotic (Invitrogen, Carlsbad, CA, USA) at 37°C, and cut into 10 mg pieces. Subsequently, the pieces were washed 3 times with PBS, centrifuged for 1 min at 1200 rpm at room temperature, and cultured overnight in adipocyte medium containing Dulbecco’s modified Eagle medium F12 supplemented with 10% fetal calf serum, 33 µmol/L biotin, 17 µmol/L panthothenate (all from Invitrogen, Carlsbad, CA, USA), and antibiotic-antimycotic, in a humidified atmosphere (95% air and 5% CO_2_) at 37°C. Then, CM was generated by culturing the explants in serum-free adipocyte medium (100 mg explants/mL) for 24 h. The CM was collected and stored as aliquots at −80°C until further use.

### Omentin-1 Levels

Omentin-1 levels in human plasma and CM were determined using an omentin-1 Enzyme Linked Immuno Sorbent Assay (ELISA; USCN Life Science Inc, Cologne, Germany). The intra- and interassay coefficients of variation for the omentin-1 ELISA were less than 6.7% and 9.1%, respectively. For protein expression, biopsies were homogenized in 50 mM Tris.HCl [pH 7.5], 150 mM NaCl, 0.5% Triton X-100, 1 mM NaF, 1 mM Na_3_VO_4_, 2 mM MgCl_2_, 1 mM DTT, and protease inhibitors (Complete, Roche Diagnostics, Mannheim, Germany). Then, homogenates were cleared by centrifugation (15 min; 12.000 rpm; 4°C), and protein content was determined using Bradford reagent (Biorad Laboratories, Munich, Germany). Protein expression was determined by Western blot analysis of ten microgram of protein using omentin-1 antibody (R&D systems, Minnesota, MN, USA). Immunoblots were quantified using a LUMI Imager system (Roche Diagnostics, Mannheim, Germany), and normalized by reprobing the stripped filters with glyceraldehyde 3-phosphate dehydrogenase (GAPDH) antibody (Abcam plc, Cambridge, UK).

### Preparation of Adult Rat Cardiomyocytes

Animal experiments were performed in accordance with the ‘Principle of laboratory animal care’ (NIH publication No. 85–23, revised 1996) and the current version of the German Law on the protection of animals, and were approved by the animal ethics committee of the Heinrich Heine University (Duesseldorf, Germany). Cardiomyocytes were isolated from male Lewis rats (Lew/Crl) weighing 150–300 gram using a Langendorff perfusion system as described [29]. Briefly, rats were killed following anaesthesia with ketamine (100 mg/kg; Ratiopharm, Ulm, Germany) and xylazine (Rompun, 5 mg/kg) (Bayer Healthcare, Leverkusen, Germany). The isolated heart was retrograde perfused using a Langendorff perfusion system and digested with a buffer containing collagenase (Worthington, Lakewood, NJ, USA) and hyaluronidase (Applichem, Darmstadt, Germany). Isolated cardiomyocytes were seeded in Medium 199 with Hank’s salts, supplemented with ITS (insulin, transferrin, selenium), 100 U/mL penicillin, 100 mg/mL streptomycin and 5% fetal calf serum (all from PAA laboratories, Pasching, Austria) on laminin-coated 35 mm culture dishes (for signaling experiments: Greiner Bio-One GmbH, Solingen, Germany; for fluorescence analysis: ibidi GmbH, Martinsried, Germany). The medium was renewed after 4 h, and culture was continued overnight.

### Analysis of Contractile Function and Insulin Signaling

To determine contractile function, isolated cardiomyocytes were cultured overnight and then preloaded with Fura-2-AM (Merck chemicals, Darmstadt, Germany) for 25 min at room temperature. Then, cultures were washed twice with adipocyte medium and incubated for 30 min with CM (diluted 1∶6 with adipocyte medium) or adipocyte medium in the presence or absence of 300 ng/mL recombinant human omentin (Cell systems GmbH, Germany; CS-C1212). Subsequently, sarcomere shortening and Ca^2+^-transients were analyzed using an IonOptix system (Milton, MA, USA) as described [3]. For analysis of Akt-phosphorylation by Western blotting, cardiomyocytes were incubated for 24 h with CM (diluted 1∶6 with adipocyte medium) or adipocyte medium in the presence or absence of 300 ng/mL recombinant human omentin. Then, cells were stimulated for 10 min with 100 nM insulin, and lysed as described [3].

### Statistical Analysis

Data are presented as mean ± standard error of the mean or median (interquartile range) in case of a non-Gaussian distribution. Significant differences between the variables were evaluated as indicated in the legends to the Figures and Tables. Correlation coefficients were calculated using the Pearson’s correlation. Linearity of the regression models was judged based on histograms and scatter plots. Because no interactions were found between group and dependent variables, both groups were analyzed as one for regression analysis. Additional potential confounders were investigated by adding group state, BMI, and M-value for insulin sensitivity for associations of omentin-1. Variables that changed the regression coefficients by more than 10% were included in the adjusted model. IBM SPSS Statistics version 20 was used for these analyses. A value of *P<*0.05 was considered as statistically significant.

## Results

### Expression and Secretion of Omentin-1 in Adipose Tissue Depots

**Figure 1:**
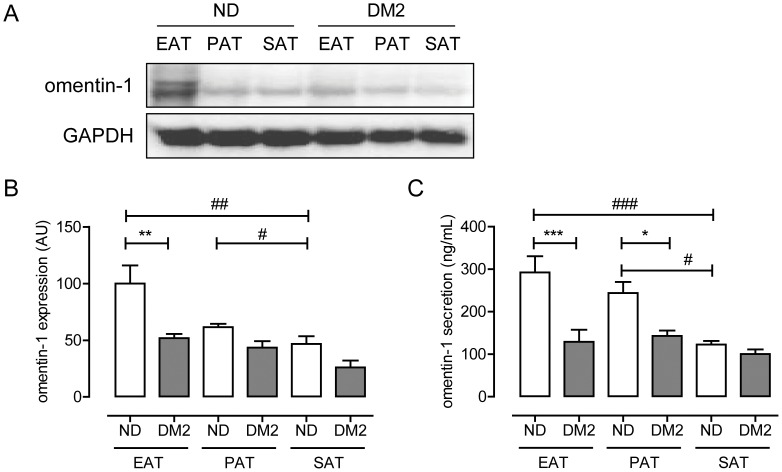
Expression and secretion of omentin-1 in intrathoracal adipose tissue depots. Representative Western blot (**A**) and quantification (**B**) of omentin-1 expression in paired epicardial (EAT), pericardial (PAT), and subcutaneous (SAT) adipose tissue biopsies of patients with (DM2, n = 7) and without (ND, n = 6) type 2 diabetes. Equal loading was verified by probing the immunoblots with glyceraldehyde 3-phosphate dehydrogenase (GAPDH) antibody. (**C**) Quantification of omentin-1 levels in conditioned media generated from paired EAT, PAT and SAT explants from DM2- and ND-patients. Data are expressed as mean ± SEM (n = 6 patients per group). ***indicates *P*<0.001; **P<0.01, *P<0.05 for differences between ND and DM2 (ANOVA followed by Bonferonni analysis for multiple comparisons); ^###^indicates *P*<0.001; ^##^ *P*<0.01, and ^#^ *P*<0.05 for differences between the various fat depots (paired *t*-test).

Omentin-1 expression was determined in adipose tissue biopsies collected from patients with and without DM2 undergoing open heart surgery. As shown in Table 1, both groups had comparable age and BMI, while fasting blood glucose levels were elevated in patients with DM2. Omentin-1 protein expression was highest in EAT from non-DM2 subjects as compared to pericardial and subcutaneous adipose tissue from the same patient, respectively (Figure 1A/B). Furthermore, omentin-1 levels in EAT were reduced in patients with DM2 versus non-DM2 patients (*P*<0.01) (Figure 1A/B). Analysis of CM showed highest omentin-1 secretion by EAT and pericardial adipose tissue as compared to subcutaneous adipose tissue in non-DM2 patients (Figure 1C). Furthermore, omentin-1 levels in CM were markedly reduced in DM2 (Figure 1C).

**Table 1:** Characteristics of patients from which adipose tissue biopsies were collected.

	ND-patients (n = 11)	DM2-patients (n = 14)
**Anthropometric parameters:**		
Age (years)	68.4±2.2	71.9±2.4
BMI (kg/m^2^)	26.9±1.3	28.9±1.1
Fasting plasma glucose (mg/dl)	96.8±11	132±20*
**Medication use:**		
lipid-lowering	7	8
diuretics	4	6
anti-hypertension	9	8
glucose-lowering	0	5*

Data are presented as mean ± SD. *P*-values for differences between anthropometric variables were calculated using the Mann-Whitney U-test in case of continuous data, and using Fisher’s exact test for medication use.

* *P*<0.05. BMI, body mass index; DM2, type 2 diabetes; ND, non-DM2.

### Circulating Omentin-1 Levels in Type 2 Diabetes and Controls

**Figure 2:**
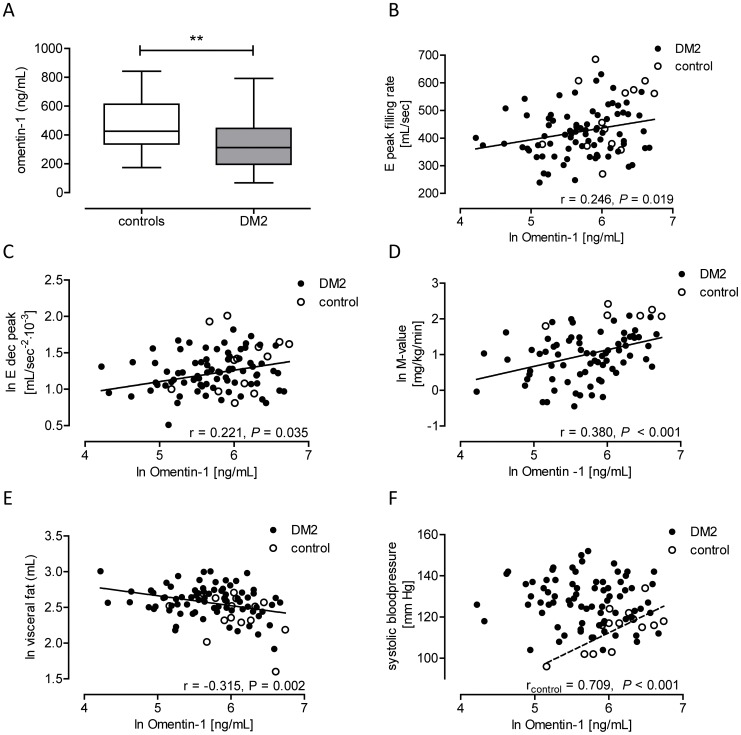
Plasma omentin-1 levels in men with uncomplicated type 2 diabetes. Plasma omentin-1 levels, fat distribution, insulin sensitivity and diastolic parameters were determined in healthy control men (n = 14) and men with uncomplicated type 2 diabetes (DM2) (n = 78). (**A**) Whisker plot (median, min-max) depicting plasma omentin-1 levels in controls and DM2-patients. Differences in circulating omentin-1 levels were analyzed using a Mann-Whitney U-test. **indicates *P*<0.01. Regression analysis identified significant correlations between baseline omentin-1 plasma levels and E peak filling rate (**B**), early deceleration peak (**C**), M-value (**D**), visceral fat volume (**E**), and systolic blood pressure (**F**). A straight line indicates a regression line for all subjects. A dashed line indicates a regression line for healthy controls only.

Plasma omentin-1 levels were determined in 78 men with uncomplicated DM2 and 14 healthy controls. Detailed baseline and cardiometabolic parameters of the participants have been described previously [23]. Briefly, patients with DM2 had a slightly higher BMI, increased systolic and diastolic blood pressure, visceral fat and subcutaneous fat volumes, as well as elevated HbA1c and fasting plasma glucose and insulin levels (all *P*<0.05) (Table 2). Furthermore, patients with DM2 versus healthy controls had a lower M-value for insulin sensitivity, impaired left ventricular diastolic function, increased pulse wave velocity, and decreased distensibility of the aorta ascendens (all *P*<0.05) (Table 2). As compared to controls, median plasma omentin-1 levels were lower in patients with DM2 (313 versus 426 ng/mL; *P* = 0.008) (Figure 2A).

**Table 2:** Characteristics of subjects for determination of circulating omentin-1 levels.

	Controls (n = 14)	DM2-patients (n = 78)
**Baseline characteristics, fat volume, insulin sensitivity**		
Age, years‡	54.5±7.1	56.5±5.6
BMI, kg/m^2^ ‡	27.0±2.5	28.7±3.5**
Disease duration, years‡	n.a.	4 (2–6)
Visceral abdominal fat volume, mL	326 (195–422)	408 (300–531)*
Subcutaneous abdominal fat volume, mL	528±30	684±258*
Pericardial fat volume, mL	26.9±10.2	29.8±9.7
M-value, mg/kg.min‡	8.1 (7.4–10.0)	2.7 (1.6–4.2)***
**Plasma parameters**		
Fasting plasma glucose, mmol/L‡	5.3 (5.0–5.6)	8.3 (7.1–10.0)***
Fasting plasma insulin, pmol/L‡	28 (19–33)	64 (36–92)***
HbA1c, %‡	5.3±0.2	7.1±1.0***
Total cholesterol, mmol/L‡	5.3±0.7	4.7±1.0***
HDL-cholesterol, mmol/L‡	1.4 (1.3–1.6)	1.1 (0.9–1.3)***
Triglycerides, mmol/L‡	0.9 (0.7–1.1)	1.5 (1.0–2.2)***
Plasma non-esterified fatty acids, mmol/L‡	0.46 (0.37–0.52)	0.50 (0.40–0.62)
**Myocardial metabolism**		
Myocardial triglyceride content, %‡	0.57 (0.43–0.85)	0.81 (0.52–1.05)#
MMRglu, nmol/ml/min‡	834.9±202.8	402.0±152.6***
MFAU, nmol/ml/min‡	55.3 (45.0–71.6)	85.7 (63.7–130.8)*
MFAO, nmol/ml/min‡	51.9 (39.4–69.9)	77,6 (61.9–103.3)**
MFAE, nmol/ml/min‡	3.42 (1.70–5.59)	0.0011 (0–2.61)#
**Hemodynamic parameters, cardiac dimensions and function**		
Systolic blood pressure, mm Hg‡	118±11	128±12***
Diastolic blood pressure, mm Hg‡	72±8	76±7*
Heart rate, beats/min‡	56 (51–62)	64 (60–70)***
Rate pressure product, (beats/min).mm Hg‡	6684±1441	8345±1457***
LV mass, gram‡	111±24	107±17
LV mass/volume ratio, gram/mL	0.62±0.09	0.70±0.11*
LV end diastolic volume, mL‡	181±56	156±25***
LV end systolic volume, mL‡	72 (63–82)	59 (52–71)**
Stroke volume, mL‡	107±23	94±16**
Ejection fraction, %‡	59±4	60±6
Cardiac output, L/min	6237±1324	6175±1160
Pulse wave velocity, m/s	5.4 (4.9–6.2)	6.3 (5.5–7.0)**
Aorta ascendens distensibility, 10^−3^ mm Hg^−1^	6.2 (4.8–7.7)	3.2 (2.3–5.9)***
E acceleration peak, mL/s^2^.10^−3^	5.9 (4.9–8.7)	6.3 (5.6–7.5)
E acceleration mean, mL/s^2^.10^−3^	4.3 (3.0–5.5)	4.0 (3.3–4.6)
E peak filling rate, mL/s‡	503±112	417±84***
E deceleration peak, mL/s^2^.10^−3^ ‡	4.73 (3.11–5.19)	3.40 (2.89–3.99)**
E deceleration mean, ml/s^2^.10^−3^	2.71±0.80	2.26±0.67*
E/A peak ratio	1.26±0.36	1.04±0.25*

Data are mean ± SD or median (interquartile range). *P*-values for differences between variables were calculated using the students *t*-test in case of normally distributed data, or the Mann-Whitney U-test in case of non-Gaussian distributions data.

*** indicates *P*<0.001;

** *P*<0.01;

* *P*<0.05;

# *P*<0.10. A, diastolic atrial contraction; BMI, body mass index; DM2, type 2 diabetes; E, early diastolic filling phase; HbA1c, glycated hemoglobin; HDL, high-density lipoprotein; LV, left ventricular; MFAE, myocardial fatty acid esterification; MFAO, myocardial fatty acid oxidation; MFAU, myocardial fattu acid uptake; MMRGlu, myocardial metabolic glucose metabolism; M-value, whole body insulin sensitivity.

‡ adapted from Rijzewijk et al. 2009 [23].

Univariate regression analysis identified inverse correlations between plasma omentin-1 levels and BMI, fasting plasma glucose, insulin and HbA1c, visceral fat volume, heart rate, and rate pressure product, and positive associations with the diastolic parameters early peak filling rate, early deceleration peak and early deceleration mean, M-value for insulin sensitivity, adiponectin and HDL-cholesterol (Table 3, Figure 2B–F). Only in controls, omentin-1 levels associated with systolic blood pressure (Table 3, Figure 2F). In multivariate regression analysis, the associations between omentin-1 plasma levels and early peak filling rate and early deceleration mean were independent of diabetic state, BMI, and M-value (respectively β = 0.247; *P* = 0.04; β = 0.255, *P* = 0.004). The association between omentin-1 plasma levels and early deceleration peak was borderline significant in this analysis (β = 0.233; *P = *0.06).

**Table 3:** Correlations between plasma omentin-1 levels and anthropometric, plasma, hemodynamic parameters, and cardiac dimensions and function.

	All subjects (n = 92)	Controls (n = 14)	DM2-patients (n = 78)
**Baseline characteristics, fat volume, insulin sensitivity**			
Age, years	−0.025	−0.061	0.005
BMI, kg/m^2^	−0.219*	−0.069	−0.184
Disease duration, years	n.a.	n.a.	0.131
Visceral abdominal fat volume, mL	−0.269**	−0.363	−0.205#
Subcutaneous abdominal fat volume, mL	−0.176#	0.037	−0.149
Pericardial fat volume, mL	−0.180#	−0.288	−0.138
M-value, mg/kg.min	0.379***	0.502	0.322**
**Plasma parameters**			
Fasting plasma glucose, mmol/L	−0.227*	0.059	−0.090
Fasting plasma insulin, pmol/L	−0.378***	0.117	−0.379***
HbA1c, %	−0.259*	−0.589#	−0.146
Total cholesterol, mmol/L	0.235*	0.703*	0.152
HDL-cholesterol, mmol/L	0.280**	0.187	0.230*
Triglycerides, mmol/L	−0.173	0.474	−0.131
Plasma non-esterified fatty acids, mmol/L	−0.050	−0.345	0.015
**Myocardial metabolism**			
Myocardial triglyceride content, %^‡^	0.156	−0.439	−0.087
MMRglu, nmol/ml/min	0.142	−0.106	0.142
MFAU, nmol/ml/min	−0.115	−0.510	−0.057
MFAO, nmol/ml/min	−0.130	−0.420	−0.064
MFAE, nmol/ml/min	−0.008	−0.128	−0.030
**Hemodynamic parameters, cardiac dimensions and function**			
Systolic blood pressure, mm Hg^‡^	−0.130	0.709**	−0.108
Diastolic blood pressure, mm Hg^‡^	−0.037	0.476	−0.113
Heart rate, beats/min^‡^	−0.221*	−0.210	−0.153
Rate pressure product, (beats/min).mm Hg^‡^	−0.243*	0.117	−0.184
LV mass, gram^‡^	−0.071	0.164	−0.147
LV mass/volume ratio, gram/mL	−0.167	0.181	−0.144
LV end diastolic volume, mL^‡^	0.076	0.007	−0.009
LV end systolic volume, mL^‡^	0.098	−0.053	0.019
Stroke volume, mL^‡^	0.039	0.104	−0.024
Ejection fraction, %^‡^	−0.074	0.179	−0.032
Cardiac output, L/min	−0.098	0.020	−0.125
Pulse wave velocity, m/s	0.031	0.067	0.060
Aorta ascendens distensibility, 10^−3^ mm Hg^−1^	0.175#	0.006	0.132
E acceleration peak, mL/s^2^.10^−3^	0.148	0.316	0.122
E acceleration mean, mL/s^2^.10^−3^	0.146	0.207	0.114
E peak filling rate, mL/s^‡^	0.246*	0.325	0.182
E deceleration peak, mL/s^2^.10^−3‡^	0.221*	0.232	0.185
E deceleration mean, mL/s^2^.10^−3^	0.218*	0.278	0.179
E/A peak ratio	0.139	0.419	0.040

Data are Pearson’s r. In case of non-Gaussian distributions, parameters were ln-transformed for correlation analysis. A, diastolic atrial contraction; BMI, body mass index; DM2, type 2 diabetes; E, early diastolic filling phase; HbA1c, glycated hemoglobin; HDL, high-density lipoprotein; LV, left ventricular; MFAE, myocardial fatty acid esterification; MFAO, myocardial fatty acid oxidation; MFAU, myocardial fattu acid uptake; MMRGlu, myocardial metabolic glucose metabolism; M-value, whole body insulin sensitivity.

# *P*<0.10;

* *P*<0.05;

** *P*<0.01;

*** *P*<0.001.

**Figure 3:**
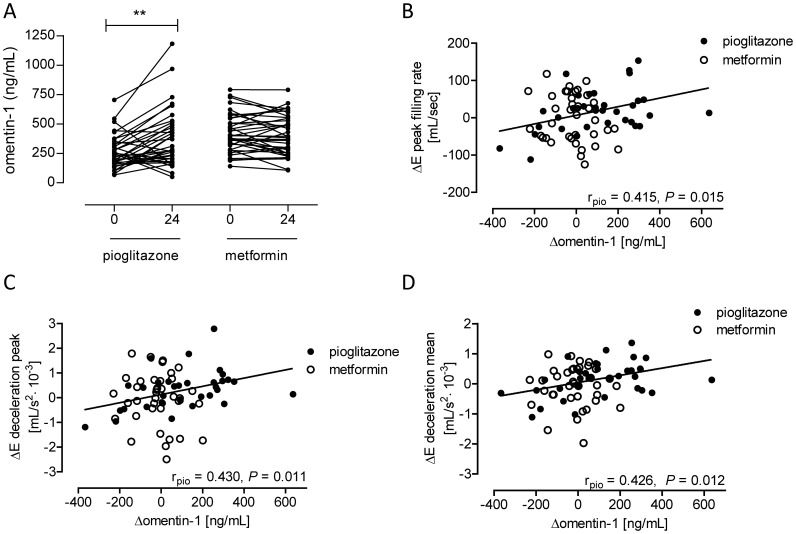
Plasma omentin-1 levels in men with uncomplicated type 2 diabetes before and after 24-week pioglitazone treatment versus 24-week metformin treatment. (**A**) Plasma omentin levels before (0) and after 24 weeks of treating males with uncomplicated type 2 diabetes with metformin or pioglitazone. *P*-values for treatment-effects were calculated using a Wilcoxon signed rank test. **indicates a *P*<0.01. Pearson regression analysis showed that only in the pioglitazone group changes in omentin-1 levels positively correlated with changes in early peak filling rate (**B**), early deceleration peak (**C**), and early deceleration mean (**D**).

Treating patients with DM2 with pioglitazone or metformin led to similar improvement of glycemic control, but only pioglitazone treatment resulted in improved left ventricular diastolic function [22]. Plasma omentin-1 levels were increased in patients with DM2 following pioglitazone (baseline versus pioglitazone: 257 versus 375 ng/mL; *P* = 0.002), but not metformin, treatment (Figure 3A). Furthermore, changes in omentin-1 levels were correlated with changes in early peak filling rate, early deceleration peak and early deceleration mean in patients with DM2 after 24-week of pioglitazone treatment, but not after metformin treatment (Figure 3B–D).

### Effect of Recombinant Omentin on Cardiomyocyte Contractile Function and Insulin Action

**Figure 4:**
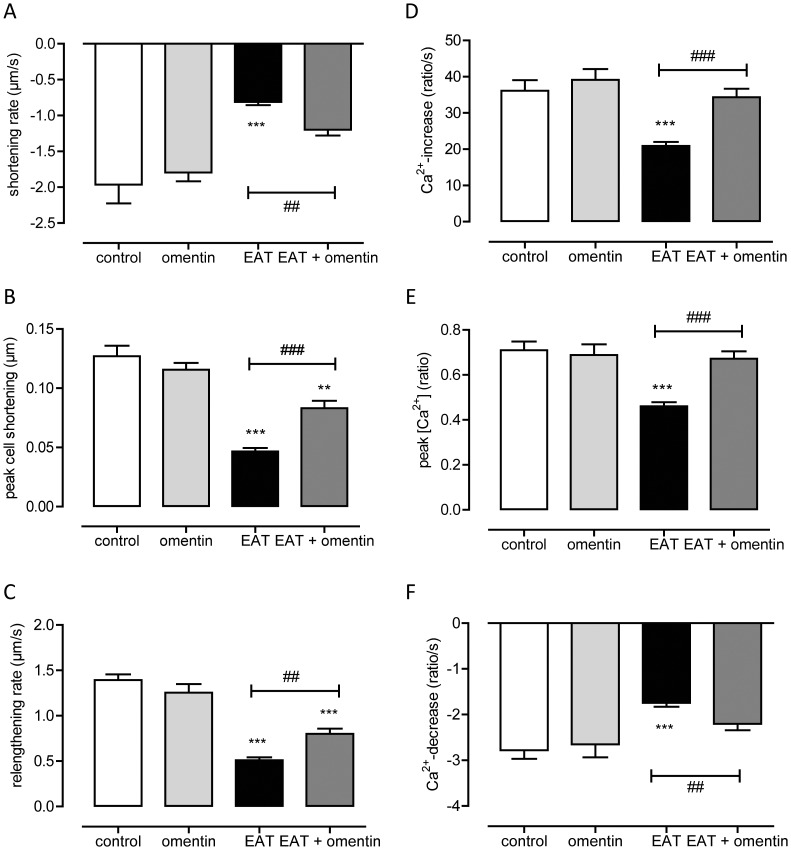
Effect of recombinant omentin on sarcomere shortening and calcium fluxes in primary adult rat cardiomyocytes. Primary rat cardiomyocytes were incubated with control medium or conditioned media from epicardial adipose tissue from patients with type 2 diabetes (EAT) for 30 min in the absence or presence of recombinant omentin before analysis of sarcomere shortening and cytosolic Ca^2+^-fluxes. Effect of exposure of cardiomyocytes to EAT and omentin on departure velocity of contraction (**A**), peak sarcomere shortening (**B**), return velocity of contraction (**C**), departure velocity of cytosolic [Ca^2+^] (**D**), peak fura-2 fluorescence (**E**) and departure velocity of cytosolic [Ca^2+^] (**F**). Data were collected during at least 4 independent experiments using cardiomyocyte preparations from different rats and conditioned media from different donors, and are expressed as mean ± standard error of the mean. Differences among the groups were evaluated using the Kruskal-Wallis method followed by a Dunns multiple comparison test. ***P<0.001; **P<0.01, versus control adipocyte medium (control), ^###^ *P*<0.001; ^##^ *P*<0.01 EAT versus EAT+omentin.

**Figure 5:**
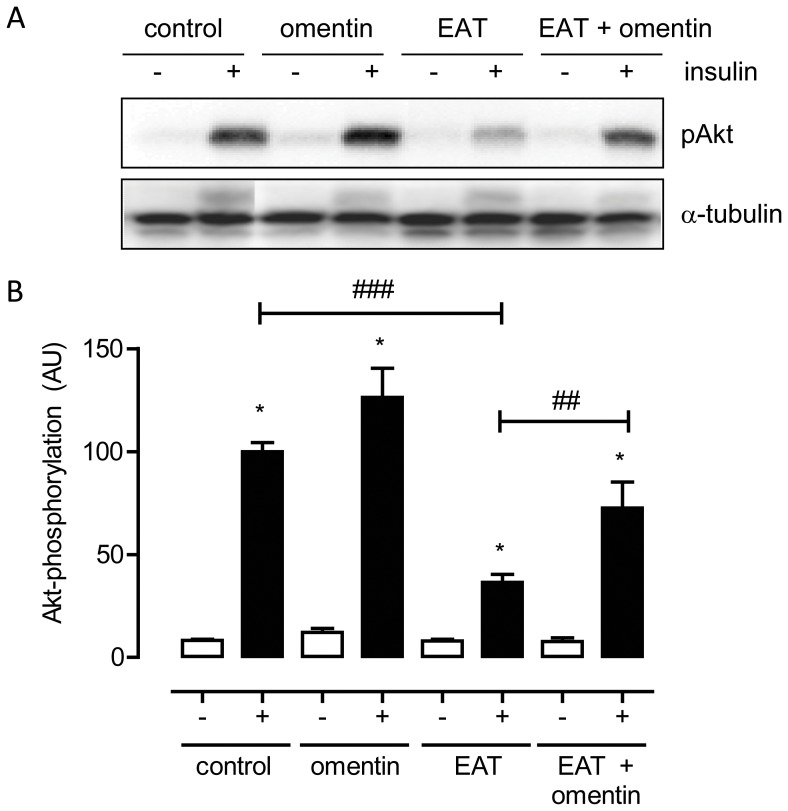
Effect of recombinant omentin on insulin action in primary adult rat cardiomyocytes. Western blot (**A**) and quantification (**B**) of recombinant omentin on insulin action. Lysates from primary adult rat cardiomyocytes exposed for 24 h to control adipocyte medium (control) or recombinant omentin in the absence or presence of conditioned media generated from epicardial adipose tissue from patients with type 2 diabetes (EAT) were analyzed for insulin-induced Akt-Ser473-phosphorylation. Data were collected during at least 6 independent experiments using cardiomyocyte preparations from different rats and conditioned media from different donors, and are expressed as mean ± standard error of the mean. Open bars, basal; filled bars, insulin stimulated cells. Differences among the groups were evaluated by ANOVA following Bonferroni analysis for multiple comparisons. *P<0.001 effect of insulin (filled bars) versus basal (open bars); ^###^ *P*<0.001 control versus EAT, ^##^ *P*<0.01 EAT versus EAT+omentin.

The above findings suggest that increases in omentin-1 levels could beneficially affect cardiac function in patients with DM2. To substantiate this, we investigated whether recombinant omentin could prevent the induction of cardiomyocyte dysfunction by CM generated from EAT from patients with DM2 (CM-EAT). Exposure of primary rat cardiomyocytes to CM-EAT reduced peak sarcomere shortening and the departure and return velocity of contraction as compared to cardiomyocytes exposed to control medium (Figure 4A–C). Omentin alone had no effect on contractile function, but partially prevented the reductions in the parameters of sarcomere shortening induced by CM-EAT (Figure 4A–C). Furthermore, the abrogation in cytosolic Ca^2+^-transients induced by CM-EAT, as illustrated by reductions in departure and return velocities and a lower peak Fura-2 fluorescence signal, was fully reversed by the presence of omentin (Figure 4D–F). Finally, we examined the effect of omentin on the reduction of insulin-stimulated Akt-phosphorylation caused by exposure of cardiomyocytes to CM-EAT. As shown in Figure 5, omentin alone had no significant effect on insulin-mediated Akt-phosphorylation. Yet, the presence of omentin prevented the abrogation of insulin-mediated Akt-phosphorylation induced by CM from patients with DM2 (Figure 5).

## Discussion

Here we show that decreases in circulating omentin-1 levels associate with left ventricular diastolic dysfunction. In patients with DM2, circulating omentin-1 levels were lower as compared to controls. Pioglitazone treatment, which improved left ventricular diastolic function in this study cohort [22] increased plasma omentin-1 levels. Furthermore, in pioglitazone-treated patients with DM2, the increments in plasma omentin-1 levels correlated positively with the observed changes in diastolic function. Finally, in cardiomyocytes, the presence of omentin prevented the induction of contractile dysfunction and insulin resistance by CM-EAT from patients with DM2. Collectively, these findings provide evidence for a cardioprotective function of omentin-1, and suggest that decreases in omentin levels may contribute to the pathogenesis of cardiac dysfunction in DM2.

This study confirmed that circulating omentin-1 levels are lower in patients with DM2 versus controls [10], [12], that positive associations exist between omentin-1 levels and insulin sensitivity, adiponectin, and high-density lipoprotein cholesterol, and that negative associations exist between omentin-1 levels and measures of adiposity, such as BMI, visceral fat volume, fasting plasma insulin and glucose levels [8], [10], [11], [13], [14]. Circulating omentin-1 levels also weakly associate with endothelial function in men with normal and impaired glucose tolerance [9], [14]. We observed a weak tendency of omentin-1 levels associating with aorta distensibility, but not with pulse wave velocity. Other studies reported increases in omentin-1 levels following a 6–month metformin intervention in women with the polycystic ovary syndrome [30], and following treatment of Chinese patients with DM2 with poor glycemic control with metformin combined with liraglutide [31]. Although metformin improved glycemic control [22], we observed no effect of metformin on omentin-1 levels in our study population containing men with well-controlled DM2. It remains to be clarified whether this can be ascribed to differences among the study cohorts, such as gender, glycemic control, disease status, ethnicity, and the lack of a ‘metformin only’ group in the Chinese study [31].

A major finding in our study is the association of plasma omentin-1 levels with left ventricular diastolic function. Because of the strong positive association with adiponectin levels, omentin-1 levels may be regulated by adiponectin [12]. Like omentin-1, adiponectin positively associates with insulin sensitivity and high-density lipoprotein cholesterol, and negatively with BMI, and waist circumference, but not with parameters of left ventricular diastolic function in this study population [32]. Although these findings do not exclude a role for adiponectin in the regulation of omentin-1, the data suggest a direct cardioprotective effect of omentin-1 rather than adiponectin-mediated.

*In vitro* studies have indeed ascribed a signaling function to omentin-1 because it promotes Akt-phosphorylation in isolated blood vessels, vascular smooth muscle cells, and microvascular endothelial cells [30], [33], [34], and enhances insulin-mediated Akt-phosphorylation and glucose uptake in adipocytes [15]. Furthermore, omentin-1 has an anti-inflammatory action as illustrated by its ability to reduce the induction of migration, angiogenesis, and activation of nuclear factor kappa B (NF-kB) and p38 by pro-inflammatory factors in endothelial cells and smooth muscle cells [16], [30]. In recent studies, we observed that factors secreted from EAT can directly affect contractile function and insulin action in primary adult rat cardiomyocytes [3], [6]. Moreover, we could show that DM2 induces qualitative alterations in the secretory profile of EAT, which may contribute to the induction of cardiac dysfunction [6]. In the present study, we demonstrated that circulating omentin-1 levels and omentin-1 released from EAT are reduced in DM2. Analysis of omentin-1 action in cardiomyocytes showed that omentin alone had no effect on sarcomere shortening, cytosolic Ca^2+^-fluxes and Akt-phosphorylation in cardiomyocytes. Rather, omentin-1 was found to protect against the induction of cardiomyocyte contractile dysfunction and insulin resistance by EAT-released factors from patients with DM2. This suggests that omentin-1 could exert its cardioprotective effects by acting as a scavenger for detrimental factors secreted by adipose tissue.

Yet, several issues remain to be addressed. First of all, this study was conducted in men, and it is unclear to what extent our findings can be applied to women. In this respect, the clinical studies that addressed this issue reported no impact of gender on circulating omentin-1 levels [35]–[38]. However, one should note that others reported conflicting data with elevated levels found in both men and women as compared to the other gender [8], [12], and sometimes dependent on disease status [31]. Furthermore, it is unclear how omentin-1 synthesis is regulated in response to external stimuli. In adipose tissue, omentin-1 is predominantly produced by the stromal vascular fraction [15], which contains a wide variety of non-adipose cells, including pre-adipocytes, endothelial cells, stem cells, fibroblasts, and immune cells. Adipose tissue in DM2 is characterized by infiltration of immune cells and an enhanced secretion of pro-inflammatory adipokines [39]. These factors may result in the induction of apoptosis and endoplasmic reticulum stress and consequently inhibition of the synthesis of abundantly expressed proteins. Finally, the beneficial effects of pioglitazone on circulating omentin-1 levels in patients with DM2 may also implicate a critical role for peroxisome proliferator activated receptor-γ in the regulation of omentin-1 production. However, because of the complex cellular composition of adipose tissue, it may be preferable to specify the cell type(s) in which omentin-1 is produced first, before studying a contribution of regulators of omentin-1 synthesis.

### Conclusions

This study shows that omentin-1 levels in plasma and EAT are decreased in patients with DM2. Furthermore, the positive association of omentin-1 levels with left ventricular diastolic function and the experiments in isolated rat cardiomyocytes suggest that omentin-1 could have a cardioprotective function. Accordingly, a reduction in omentin-1 expression in EAT might contribute to the induction of cardiac dysfunction in patients with DM2.
